# Aetiology and Antibiotic Susceptibility of Common Bacterial Infections in Hospitalised Patients: A 2019 Multisite Cross‐Sectional Survey in Jakarta, Indonesia

**DOI:** 10.1111/tmi.70162

**Published:** 2026-05-29

**Authors:** Justin de Brabander, Erni J. Nelwan, Ralalicia Limato, Monik Alamanda, Manzilina Mudia, Enty Tjoa, Ifael Y. Mauleti, Maria Mayasari, Iman Firmansyah, T. Mannaria Jayati, Michèle van Vugt, H. Rogier van Doorn, Raph L. Hamers, Erni J. Nelwan, Ralalicia Limato, Manzilina Mudia, Monik Alamanda, Helio Guterres, Enty Enty, Y. Ifael, Maria Mayasari Mauleti, Iman Firmansyah, May Hizrani, Anis Karuniawati, Taralan Tambunan, Amin Soebandrio, Decy Subekti, Iqbal Elyazar, Mutia Rahardjani, Fitria Wulandari, Reinout van Crevel, H. Rogier van Doorn, Vu Thi Lan Huong, Nga Do Ti Thuy, Sonia Lewycka, Alex Broom, Raph L. Hamers

**Affiliations:** ^1^ Faculty of Medicine Oxford University Clinical Research Unit Indonesia, Universitas Indonesia Jakarta Indonesia; ^2^ Department of Internal Medicine, Division of Infectious Diseases Amsterdam UMC, University of Amsterdam Amsterdam the Netherlands; ^3^ Faculty of Medicine Universitas Indonesia Jakarta Indonesia; ^4^ Department of Internal Medicine, Division of Infectious Diseases Cipto Mangunkusumo National General Hospital Jakarta Indonesia; ^5^ Infectious Disease and Immunology Research Cluster, Indonesian Medical Education and Research Institute Jakarta Indonesia; ^6^ Metropolitan Medical Centre Hospital Jakarta Indonesia; ^7^ Centre for Tropical Medicine and Global Health, Nuffield Department of Medicine University of Oxford Oxford UK; ^8^ Royal Taruma Hospital Jakarta Indonesia; ^9^ School of Medicine and Health Sciences, Atma Jaya Catholic University Jakarta Indonesia; ^10^ Fatmawati General Hospital Jakarta Indonesia; ^11^ St. Carolus Hospital Jakarta Indonesia; ^12^ Prof. Dr. Sulianti Saroso Infectious Disease Hospital Jakarta Indonesia; ^13^ Oxford University Clinical Research Unit Hanoi Vietnam

**Keywords:** antimicrobial resistance, bacterial pathogens, community‐acquired infection, hospital‐acquired infection, hospitals, Indonesia, point prevalence survey

## Abstract

**Objectives:**

To describe the aetiology and antibiotic susceptibility patterns of common bacterial infections in hospitalised patients in Jakarta, Indonesia.

**Methods:**

We conducted a hospital‐wide point prevalence survey in six hospitals in 2019, capturing data from routinely ordered bacterial cultures taken before the start of antibiotic treatment. We report relevant bacteria‐antibiotic combinations for *Escherichia coli* and the ESKAPE group of pathogens.

**Results:**

A total of 562 patients (52% women, median age 46 years) were diagnosed with 587 infections, comprising 414 community‐acquired and 173 hospital‐acquired infections, with pneumonia most frequent (258, 44%). From 615 samples collected, 279 (45%) bacterial isolates were identified, of which 213 (76%) gram‐negative, including *Klebsiella pneumoniae* (68, 24%), *Escherichia coli* (37, 13%) and *Pseudomonas aeruginosa* (35, 13%) among others, and 66 (24%) gram‐positive, including *Enterococcus faecalis* (17, 6%) and *Staphylococcus aureus* (11, 4%) among others. Proportions of bacteria resistant to third‐generation cephalosporins (3GC) were 75% (47/63) for *Klebsiella pneumoniae* , and 69*%* (22/32) for *E. coli* , and to carbapenems 64% (7/11) for *Acinetobacter* spp., 60% (3/5) for *Enterobacter* spp., 43% (3/7) for *E. coli,* 30% (7/23) for *P. aeruginosa* and 29% (9/31) for *K. pneumoniae* . Four of 11 *S. aureus* isolates were methicillin‐resistant. A blood culture was done in only 52% (16/31) of sepsis patients, and the results of positive blood cultures were reported after a median of 4 days.

**Conclusions:**

This survey identified that a high proportion of common gram‐negative bacteria exhibited reduced susceptibility to first‐choice antibiotics and considerable underuse of bacterial cultures. These findings warrant enhanced infection prevention and control and antimicrobial stewardship.

## 1 Introduction

Antimicrobial resistance (AMR) is one of the greatest threats to global public health, with the increasing use of antimicrobial agents being one of the key drivers. A recent global analysis estimated that antibiotic‐resistant bacterial infections directly caused 1.14 million deaths and were associated with 4.71 million deaths in 2021 alone, with disproportionate impacts on low‐ and middle‐income countries (LMICs) [1].

Understanding the burden and drivers of AMR in Southeast Asia, a hotspot for AMR, is crucial for AMR control on a global scale [2, 3]. Indonesia is a middle‐income country with the largest economy and population (275 million) of the region. A range of complex factors, including variable access to quality health care, persistently high infectious disease burdens and weakly enforced antibiotic policies, render Indonesia particularly vulnerable to AMR [4, 5]. The implementation of the National Action Plan for AMR has been limited to date due to, among other factors, a limited evidence base of AMR epidemiology and antibiotic use. There is a dearth of systematic data to reliably estimate the extent of AMR, which is essential to inform evidence‐based policies to contain AMR [4, 6]. A recent systematic review of the published literature estimated that AMR levels in hospitals in Indonesia were high for critical gram‐negative bacteria, but also concluded that there were substantial data gaps, precluding robust conclusions about the full contemporary AMR situation [7].

In 2019, we conducted a point prevalence survey in six hospitals in Jakarta, Indonesia, to describe patterns of antibiotic prescribing and AMR in routine clinical practice [8]. Here, we report a ‘snapshot’ observation of the clinically relevant bacterial pathogens isolated from hospitalised patients, their antibiotic susceptibility, as well as on the uptake of bacterial cultures to contextualise these microbiological data.

## 2 Materials and Methods

### 2.1 Study Design and Population

We conducted a hospital‐wide cross‐sectional point prevalence survey in six hospitals in Jakarta between March and August 2019, using the Global‐PPS protocol, as previously described [8]. The present analysis included all inpatients of any age, who were admitted on the ward before 8AM on the day of the survey, and who started systemic antibiotic treatment (ATC J01) for a suspected bacterial infection, in accordance with local prescribing guidelines and/or physician decision. Patients receiving antibiotics solely for prophylaxis or for other or unknown reasons were excluded. We followed the STrengthening the Reporting of OBservational studies in Epidemiology (STROBE) checklist to report the study.

### 2.2 Study Setting

The participant hospitals were purposively sampled to achieve diversity in geographic location, size, ownership and care level. The six participant hospitals were situated across all five districts of Jakarta province and comprised two tertiary care government hospitals and four secondary hospitals, of which three were privately owned and three were public, with a total of 2358 active inpatient beds (median 230, range 134–853 per hospital) (Table 1). The survey included all 238 inpatient wards (87 medical, 31 surgical, 95 mixed medical‐surgical wards and 25 ICUs, of which there were 123 adult, 51 paediatric‐neonatal and 64 mixed adult‐paediatric‐neonatal wards). At the time of the survey, all six hospitals had an on‐site microbiological laboratory service using automated culture machines and an antimicrobial stewardship programme in place, albeit at variable stages of implementation. Five of the six hospitals had local antibiotic prescribing guidelines, which did not include specific guidance on when to order a bacterial culture.

**TABLE 1:** Hospital characteristics.

	Total	Hospital 1	Hospital 2	Hospital 3	Hospital 4	Hospital 5	Hospital 6
Level of health service	—	Secondary	Tertiary	Secondary	Secondary	Tertiary	Secondary
Sector	—	Private	Public	Private	Public	Public	Private
Teaching hospital	—	Yes	Yes	Yes	No	Yes	No
National health insurance^a^	—	No	Yes	Yes	Yes	Yes	No
Hospital antibiotic guidelines	—	No	Yes	Yes	Yes	Yes	Yes
Inpatient wards^b^	238	19	74	30	14	79	22
Medical wards	87	4	27	15	6	30	5
Surgical wards	31	0	17	2	0	11	1
Mixed medical‐surgical wards^c^	95	12	19	10	7	32	15
Intensive care units	25	3	11	3	1	6	1
Adult	123	8	55	13	2	39	6
Paediatric and/or neonatal	51	5	12	6	3	21	4
Mixed adult‐neonatal‐paediatric^d^	64	6	7	11	9	19	12
Inpatient beds	2358	159	767	300	145	853	134
Admitted patients	1602 (67.9)	100 (62.9)	562 (73.3)	198 (66.0)	66 (45.5)	625 (73.3)	51 (38.1)
Admitted patients on ≥ 1 antimicrobials	993 (62.0)	75 (75.0)	368 (65.5)	106 (53.5)	52 (78.8)	359 (57.4)	33 (64.7)

*Note:* Data shown reflect the hospital situation on the survey day and are expressed as number (percentage), unless otherwise specified.

^a^ Included in the national health insurance scheme, Jaminan Kesehatan Nasional (JKN).

^b^ Includes all inpatient wards in the hospital.

^c^ Wards that can admit both medical and surgical patients.

^d^ Wards that can admit adult, paediatric and neonatal patients.

### 2.3 Data Collection and Definitions

A study team extracted patient data on medical history, working diagnosis/syndrome (as documented on the survey day) and microbiological results from routine medical and laboratory records into a standard case report form. Infections were categorised as community‐acquired (symptoms or cultures < 48 h from admission, CAI) or hospital‐acquired (symptoms or cultures ≥ 48 h after admission, HAI). All culture specimens taken as part of routine clinical care during their current admission (including < 7 days prior) before the start of antibiotic treatment were recorded.

### 2.4 Data Analysis

The analysis focused on the most relevant bacteria for hospital settings, that is, *Enterococcus* species, *Staphylococcus aureus* , *Klebsiella pneumoniae* , *Acinetobacter baumannii* , *Pseudomonas aeruginosa* and *Enterobacter* species (ESKAPE group) plus *Escherichia coli* , and common bacteria‐antibiotic combinations (including those in the WHO priority pathogen list [9]). The hospitals used the most recent Clinical and Laboratory Standards Institute (CLSI) AST interpretative criteria [10]. Intermediate susceptibility, where reported by the hospital laboratory, was considered resistant. Due to the cross‐sectional study design, we were unable to definitively determine whether the isolated pathogens were causative or contaminant. We therefore decided to include all cultured pathogens. For patients with more than one of the same pathogen per sample type, only the first isolate was included (deduplication). Where AST results were given for more than one antibiotic of the same class (e.g., ciprofloxacin and levofloxacin), the rate that reflected the highest level of resistance was selected to represent the rate for that respective class (e.g., fluoroquinolones), except for the aminoglycosides, which were reported individually, since gentamicin and amikacin show markedly different resistance rates. *S. aureus* resistant to cefoxitin and/or oxacillin were considered methicillin‐resistant *S. aureus* (MRSA).

The data were summarised as counts (percentage of total), mean (± SD) or median [IQR]. Differences between subgroups were analysed using the Pearson's chi‐squared test, unpaired *t*‐test or Mann–Whitney U test, whichever was appropriate. *p* values of < 0.05 were considered statistically significant. All statistical analyses were performed using R version 4.3.0.

## 3 Results

### 3.1 Patient Characteristics

Among 1602 hospitalised patients, 562 (35.1%) received one or more systemic antibiotics for the treatment of a presumed bacterial infection and were included in the analysis (Figure S1). The overall median age was 46 years (IQR 20–60), and 52.1% were women (Table 2). 33.8% of patients had been hospitalised and 23.8% had undergone surgery in the 3 months prior to admission. Malnutrition (200, 35.6%) and diabetes mellitus (20.6%) were common comorbidities. We recorded a total of 587 distinct infection diagnoses, comprising 414 CAI and 173 HAI. The most frequent was pneumonia (44.0%, 258), followed by skin and soft tissue (SST) (12.6%, 74), gastrointestinal (10.9%, 64), urinary tract (UTI) (7.7%, 45), intra‐abdominal infections (5.5%, 32) and sepsis without an identified focus (5.3%, 31). We recorded a total of 777 prescriptions for systemic antibiotics, comprising 542 (69.8%) for CAI and 235 (30.2%) for HAI.

**TABLE 2:** Participant characteristics.

	All participants (*n* = 562)	Patients without culture (*n* = 347)	Patients with culture (*n* = 215)	*p*	Patients with positive culture (*n* = 152)	Patients without positive culture (*n* = 63)	*p*
Age, years	46 [20–60]	49 [28–64]	38 [5–57]	< 0.001	38 [8–57]	38 [0–55]	0.37
Female	293 (52.1)	172 (49.6)	121 (56.3)	0.14	89 (58.6)	32 (50.8)	0.37
Weight, kg	52 [39–62]	55 [45–64]	49 [12–60]	< 0.001	50 [19–61]	44 [7–59]	0.05
National health insurance	423 (75.3)	244 (70.3)	179 (83.3)	0.001	127 (83.6)	52 (82.5)	> 0.99
Catheters							
Peripheral vascular catheter	534 (95.0)	336 (96.8)	198 (92.1)	0.020	139 (91.5)	59 (93.7)	0.79
Urinary catheter	189 (33.6)	91 (26.2)	98 (45.6)	< 0.001	76 (50.0)	22 (34.9)	0.06
Central vascular catheter	91 (16.2)	30 (8.6)	61 (28.4)	< 0.001	49 (32.2)	12 (19.0)	0.07
Intubation catheter	47 (8.4)	12 (3.5)	35 (16.3)	< 0.001	26 (17.1)	9 (14.3)	0.76
Recent medical history							
Surgery ≤ 90 days	134 (23.8)	50 (14.4)	84 (39.1)	< 0.001	66 (43.4)	18 (28.6)	0.06
Hospitalisation ≤ 90 days	190 (33.8)	87 (25.1)	103 (47.9)	< 0.001	72 (47.4)	31 (49.2)	0.92
Comorbidities							
Malnutrition	200 (35.6)	114 (32.9)	86 (40.0)	0.10	59 (38.8)	27 (42.9)	0.69
Diabetes mellitus	116 (20.6)	81 (23.3)	35 (16.3)	0.06	28 (18.4)	7 (11.1)	0.26
Tuberculosis	49 (8.7)	31 (8.9)	18 (8.4)	0.94	9 (5.9)	9 (14.3)	0.08
HIV	14 (2.5)	9 (2.6)	5 (2.3)	> 0.99	5 (3.3)	0 (0.0)	0.34
COPD	6 (1.1)	2 (0.6)	4 (1.9)	0.31	3 (2.0)	1 (1.6)	> 0.99
Clinical diagnosis							
Pneumonia	249 (44.3)	154 (44.4)	95 (44.2)	> 0.99	64 (42.1)	31 (49.2)	0.42
Skin and soft tissue infection	71 (12.6)	39 (11.2)	32 (14.9)	0.26	28 (18.4)	4 (6.3)	0.040
Gastrointestinal infection	63 (11.2)	55 (15.9)	8 (3.7)	< 0.001	5 (3.3)	3 (4.8)	0.90
Urinary tract infection	40 (7.1)	20 (5.8)	20 (9.3)	0.16	19 (12.5)	1 (1.6)	0.024
Sepsis without clear focus	28 (5.0)	14 (4.0)	14 (6.5)	0.27	8 (5.3)	6 (9.5)	0.40
Other diagnosis	111 (19.8)	65 (18.7)	46 (21.4)	0.51	28 (18.4)	18 (28.6)	0.14

*Note:* Data are expressed as *n* (%) or median [interquartile range].

### 3.2 Use and Reporting of Bacterial Cultures

Patients with one or more samples for bacterial culture taken were younger, more frequently had national health insurance, one or more catheters, and a recent history of surgery or hospitalisation compared to those without samples taken. Patients who had one or more positive culture results more frequently had one or more catheters and a history of recent surgery and were more frequently diagnosed with an SST or UTI, compared to those without a positive culture (Table 2).

Overall, 38.3% (215/562) of the patients underwent culture sampling (Figure S2), including in 39.9% (103/258) of pneumonia patients (sputum in 24.8% [64/358] and blood in 20.5% [53/358]); 44.6% (33/74) of SST infection patients (wound in 25.7% [19/74], blood in 14.9% [11/74] and pus in 13.5% [10/74]); 51.1% (23/45) of UTI patients (urine in 44.4% [20/45] gand blood in 33.3% [15/45]); and 54.8% (17/31) of sepsis patients (blood in 51.6% [16/31]). The median time from specimen collection to final reporting of positive results (both ID and AST results) was 4 [IQR 3–4] days for blood, 3 [2–4] days for sputum and 3 [2, 3] days for urine (Figure S3).

### 3.3 Bacterial Isolates

**FIGURE 1:**
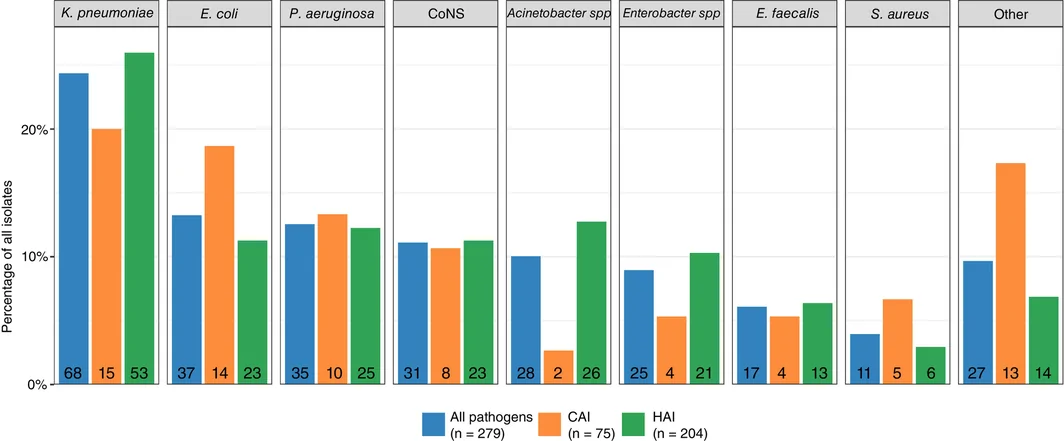
Distribution of the identified bacterial isolates, by community‐acquired and hospital‐acquired infection. Description Figure 1. The numbers in bars indicate the number of isolates. The category ‘Other’ comprised *Serratia marcescens* (*n* = 5), *Klebsiella oxytoca* , *Proteus mirabilis* , viridans group streptococci (each *n* = 3), *Vibrio vulnificus* , *Moraxella catarrhalis* , *Micrococcus luteus* (each *n* = 2) and *Stenotrophomonas maltophilia* , *Citrobacter freundii* , *Streptococcus mitis* , *Pseudomonas putida* , *Enterococcus avium* , *Morganella morganii* and *Proteus vulgaris* (each *n* = 1). CAI, community‐acquired infection; CoNS, coagulase‐negative staphylococci; HAI, hospital‐acquired infection.

In total, 279 bacterial isolates were identified from 615 specimens, including 75 isolates detected from 158 specimens in CAI and 204 isolates detected from 457 specimens in HAI (Figure 1). The total proportion of positive blood cultures was 17.9% (41/229). Overall, 76.3% (213/279) of isolates were gram‐negative bacteria (70.7% [53/75] in CAI and 78.4% [160/204] in HAI), and 23.7% (66/279) of isolates were gram‐positive bacteria (33.3% [22/66] in CAI and 66.7% [44/66] in HAI). The most frequently identified bacteria were *K. pneumoniae* (24.3%, 68), *E. coli* (13.3%, 37), *P. aeruginosa* (12.5%, 35), coagulase‐negative staphylococci (CoNS) (11.1%, 31) and *Acinetobacter* spp. (10.0%, 28), of which 15 were identified as *A. baumannii* (Figure 1). *Enterococcus faecium* was not isolated. Compared to the private hospitals, in government hospitals, *K. pneumoniae* was more frequently detected (63/247 vs. 5/32) and *Acinetobacter* spp. was less frequently detected (21/247 vs. 7/32) (Figure S4).

Pneumonia was the most common diagnosis (44%, 258/587), with the most frequent bacterial isolates being *K. pneumoniae* (18.2% [2/11] in CAI and 35.3% [30/85] in HAI), *Acinetobacter* spp. (18.2% [2/11] in CAI and 20.0% [17/85] in HAI) and *P. aeruginosa* (27.3% [3/11] in CAI and 17.6% [15/85] in HAI). For UTIs, the most frequently cultured pathogens were *E. coli* (37.5% [6/16] in CAI and 27.6% [8/29] in HAI), *E. faecalis* (12.5% [2/16] in CAI and 34.5% [10/29] in HAI) and CoNS (12.5% [2/16] in CAI and 10.3% [3/29] in HAI).

### 3.4 Antibiotic Susceptibility

**FIGURE 2:**
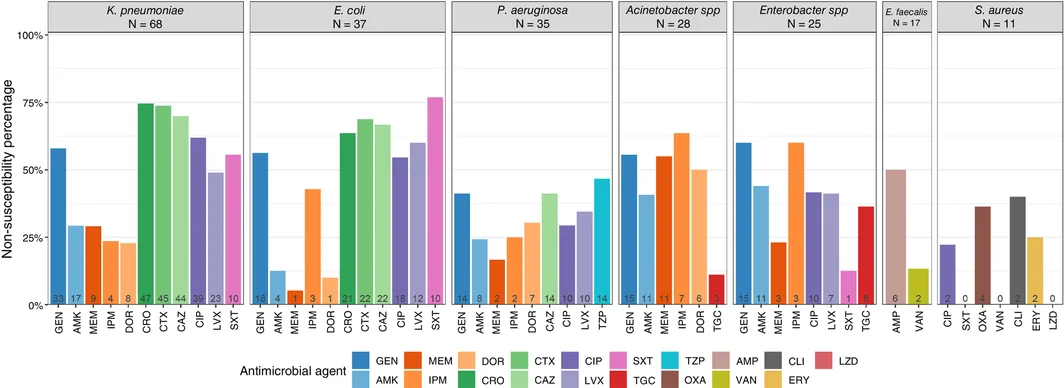
Proportion of resistance to the relevant antimicrobial agents among the identified isolates. AMK, amikacin; AMP, ampicillin; CAZ, ceftazidime; CIP, ciprofloxacin; CLI, clindamycin; CRO, ceftriaxone; CTX, cefotaxime; DOR, doripenem; ERY, erythromycin; GEN, gentamicin; IPM, imipenem; LVX, levofloxacin; LZD, linezolid; MEM, meropenem; OXA, oxacillin; SXT, trimethoprim‐sulphamethoxazole; TGC, tigecycline; TZP, piperacillin/tazobactam; VAN, vancomycin.

Among *K. pneumoniae* isolates, resistance to 3GC was detected in 47 of 63 (75%) isolates, to carbapenems in 9 of 31 (29%), fluoroquinolones in 39 of 63 (62%) and cotrimoxazole in 10 of 18 (56%) (Figure 2, Table S1,S2). Among *P. aeruginosa* isolates, resistance was detected to ceftazidime in 14 of 34 (41%), carbapenems in 7 of 23 (30%), fluoroquinolones in 10 of 29 (34%) and amikacin in 8 of 33 (24%). Among *E. coli* isolates, resistance was detected to 3GC in 22 of 32 (69%), carbapenems in 3 of 7 (43%), fluoroquinolones in 12 of 20 (60%), gentamicin in 18 of 32 (56%), amikacin in 4 of 32 (13%) and cotrimoxazole in 10 of 13 (77%). Among *Acinetobacter* spp. isolates, resistance to carbapenems was detected in 6 of 12 (50%) isolates, for which susceptibility results were available. Among *Enterobacter* spp. isolates, resistance was detected to carbapenems in 3 of 5 (60%), fluoroquinolones in 10 of 24 (42%), gentamicin in 15 of 25 (60%), amikacin in 11 of 25 (44%) and cotrimoxazole in 1 of 8 (13%). Among *S. aureus* isolates, resistance was detected to methicillin in 4 of 11 (36%) and vancomycin in 0 of 11 (0%). Among *E. faecalis* isolates, resistance was detected to amoxicillin in 6 of 12 (50%) and vancomycin in 2 of 15 (32%).

## 4 Discussion

This cross‐sectional survey conducted in six Indonesian hospitals in 2019 documented high AMR levels in gram‐negative bacteria, among the highest reported in Southeast Asia [11, 12, 13] and substantially higher than reports from most of Europe [14]. Resistance to 3GC was particularly high in *K. pneumoniae* and *E. coli* isolates, and to carbapenems in *K. pneumoniae* , *P. aeruginosa* , *E. coli* , *Acinetobacter* spp. and *Enterobacter* spp. isolates. It should be noted, however, that the numbers for some of the pathogens were too small to draw any firm conclusions, and larger, more representative surveys are required to confirm these preliminary findings.

For contextualisation of these estimates, a recent systematic review of the published literature (2000–2023), comprising 13,647 bacterial isolates from hospitals, found that in Indonesian hospitals 21.6% of *Klebsiella pneumoniae* isolates, 18.3% of *E. coli* isolates, 35.8% of *P. aeruginosa* isolates and 70.7% of *A. baumannii* isolates were carbapenem‐resistant; 29.9% of *S. pneumoniae* isolates were penicillin‐resistant; and 22.2% of *S. aureus* isolates were methicillin‐resistant. Hospital prevalence of carbapenem‐resistant *K. pneumoniae* and *E. coli* and penicillin‐resistant *S. pneumoniae* increased over time. Those estimates are aligned with two complementary reports describing routine culture data for priority bacterial pathogens from sentinel hospitals (GLASS submission, 20 hospitals and Indonesian Society for Clinical Microbiology, 75 hospitals, both from 2022) [6, 15]. The estimates available for Indonesia are among the highest in the Southeast Asia region, where nationally representative surveys of hospitalised patients have reported particularly high levels of AMR in gram‐negative bacteria and *S. aureus* in Malaysia [11], Vietnam [12] and the Philippines [13].

International guidelines on sepsis management have been stressing the importance of obtaining blood cultures before or, when not possible, within 24 h after administration of antibiotics [16]. Nonetheless, blood culture sampling rates are generally low, both in high‐income countries and LMICs, with wide variations in reported rates between hospitals and regions. Reported hospital blood culture sampling rates ranged from 196 to 308 per 1000 patient‐days in the United States [17], from 6.7 to 86.5 per 1000 patient‐days in the European Union [17] and 31, 82 and 10 per 1000 patient‐days in selected hospitals in Indonesia, Thailand and Vietnam, respectively [18, 19, 20]. In this survey, blood cultures were taken in just over half of the sepsis patients, and positive blood culture results often reached the treating clinicians with delays, hampering clinical decision‐making on targeting, de‐escalating or stopping antibiotics. Barriers in Indonesia may be a combination of factors including cost‐prohibitive bacterial culture testing and thresholds of national health insurance coverage, absence of clear culture guidelines, as well as long turnaround time and/or lack of trust in the laboratory results. We observed an association between having a positive culture result and presence of a urine catheter or recent surgery. These factors likely reflect a higher risk of infection, especially of nosocomial origin, due to invasive procedures and higher risk of antimicrobial‐resistant colonising bacteria, among these patient subgroups.

The study has some limitations. First, the limited, convenient sample of hospitals surveyed precluded generalising the findings to other hospitals or the country situation. More granular AMR data are required to better estimate the extent of the AMR problem at both the national and hospital levels, and across the spectrum from tertiary to peripheral facilities. Second, underuse of cultures in the surveyed hospitals may have limited the applicability of the data, and a prospective case‐based approach could capture more representative and informative data to inform treatment guidelines. In this regard, we note that in the Indonesian setting, culture samples are more likely obtained from hospitalised patients who are severely ill, pre‐exposed to antibiotics and at risk of a hospital‐acquired infection. This may have led to an overestimation of AMR prevalence. Third, due to the cross‐sectional study design, we were unable to determine with certainty whether all isolated pathogens were the causative agents or contaminants. The high proportion of CoNS isolated (11%) might suggest suboptimal culture sampling procedures in current practice. Lastly, our reliance on routine laboratory interpretation to classify isolates as drug‐susceptible versus resistant, without access to data on the minimal inhibitory concentrations, may have introduced heterogeneity in interpretation.

In conclusion, available data suggest that the management of severe bacterial infections in Indonesian hospitals is increasingly dependent on more expensive and less readily available antibiotics. However, there is a need for representative, more granular AMR surveillance data to accurately estimate AMR levels in key pathogens. Those efforts should be combined with rigorous implementation science focused on the implementation of effective context‐specific infection prevention and diagnostic and antibiotic stewardship interventions to improve rational antibiotic use and help mitigate AMR. 

## Funding

This work and R.L.H. and H.R.v.D. are supported by the Wellcome Africa Asia Programme Vietnam (106680/Z/14/Z). R.L. is supported by an OUCRU Prize Studentship and a Nuffield Dept of Medicine Tropical Network Fund DPhil Bursary.

## Ethics Statement

The study adhered to the Declaration of Helsinki and was approved by the Research Ethics Committee of the Faculty of Medicine Universitas Indonesia (1364/UN2.F1/ETIK/2018) and the Oxford Tropical Research Ethics Committee (559‐18). The requirement for individual informed consent was waived by the ethics committees that approved the study.

## Conflicts of Interest

The authors declare no conflicts of interest.

## Supporting information


**Table S1:** Proportion of resistance among gram‐negative isolates overall.
**Table S2:** Proportion of resistance among gram‐positive isolates overall.
**Table S3:** Proportion of resistance among gram‐negative isolates in community‐acquired infections.
**Table S4:** Proportion of resistance among gram‐positive isolates in community‐acquired infections.
**Table S5:** Proportion of resistance among gram‐negative isolates in hospital‐acquired infections.
**Table S6:** Proportion of resistance among gram‐positive isolates in hospital‐acquired infections.
**Figure S1:** Study flowchart.
**Figure S2:** Proportions of patients in whom one or more bacterial cultures were performed, by clinical syndrome.
**Figure S3:** Number of days from sample collection to reporting positive culture results, by specimen type.
**Figure S4:** Percentage of isolated bacteria, stratified by hospital ownership.

## Data Availability

The data that support the findings of this study are available on request from the corresponding author. The data are not publicly available due to privacy or ethical restrictions.
