# Supplementary material for: Aetiology and Antibiotic Susceptibility of Common Bacterial Infections in Hospitalised Patients: A 2019 Multisite Cross‐Sectional Survey in Jakarta, Indonesia
Source: Trop Med Int Health. 2026 May 29;31(9):1231–7. doi: 10.1111/tmi.70162 (PMC13533636; doi:10.1111/tmi.70162)
Supplement: Supplementary file 1 [file TMI-31-1231-s001.docx]

**Online data supplement**

**Etiology and antibiotic susceptibility of common bacterial infections in hospitalized patients: a 2019 multisite cross-sectional survey in Jakarta, Indonesia**

Justin de Brabander, Erni J. Nelwan, Ralalicia Limato, Monik Alamanda, Manzilina Mudia, Enty Tjoa, Ifael Y. Mauleti, Maria Mayasari, Iman Firmansyah, T. Mannaria Jayati, Michèle van Vugt, H. Rogier van Doorn and Raph L. Hamers

**Table S1:** Proportion of resistance among gram-negative isolates overall.

|  | **Aminoglycosides** | | **Carbapenems** | | | **3^rd^ generation cephalosporins** | | | **Fluoroquinolones** | | **Sulfo-**  **namides** | **Glycyl-**  **cyclins** | **Penicillins** |
| --- | --- | --- | --- | --- | --- | --- | --- | --- | --- | --- | --- | --- | --- |
|  | GEN | AMK | MEM | IPM | DOR | CRO | CTX | CAZ | CIP | LVX | SXT | TGC | TZP |
| *K. pneumoniae* | |  |  |  |  |  |  |  |  |  |  |  |  |
| Overall | 58 (33/57) | 29 (17/58) | 29 (9/31) | 24 (4/17) | 23 (8/35) | 75 (47/63) | 74 (45/61) | 70 (44/63) | 62 (39/63) | 49 (23/47) | 56 (10/18) |  |  |
| Blood | 63 (5/8) | 13 (1/8) | 17 (1/6) | 25 (1/4) | 20 (1/5) | 78 (7/9) | 78 (7/9) | 78 (7/9) | 56 (5/9) | 29 (2/7) | 67 (2/3) |  |  |
| Other | 57 (28/49) | 32 (16/50) | 32 (8/25) | 23 (3/13) | 23 (7/30) | 74 (40/54) | 73 (38/52) | 69 (37/54) | 63 (34/54) | 53 (21/40) | 53 (8/15) |  |  |
| *P. aeruginosa* | |  |  |  |  |  |  |  |  |  |  |  |  |
| Overall | 41 (14/34) | 24 (8/34) | 17 (2/12) | 25 (2/8) | 30 (7/23) |  |  | 41 (14/34) | 29 (10/34) | 34 (10/29) |  |  | 47 (14/30) |
| Blood | 0 (0/2) | 0 (0/2) | 0 (0/0) | 0 (0/0) | 0 (0/2) |  |  | 0 (0/2) | 0 (0/2) | 0 (0/2) |  |  | 50 (1/2) |
| Other | 44 (14/32) | 25 (8/32) | 17 (2/12) | 25 (2/8) | 33 (7/21) |  |  | 44 (14/32) | 31 (10/32) | 37 (10/27) |  |  | 46 (13/28) |
| *E. coli* |  |  |  |  |  |  |  |  |  |  |  |  |  |
| Overall | 56 (18/32) | 13 (4/32) | 5 (1/19) | 43 (3/7) | 10 (1/10) | 64 (21/33) | 69 (22/32) | 67 (22/33) | 54 (18/33) | 60 (12/20) | 77 (10/13) |  |  |
| Blood | 0 (0/2) | 0 (0/2) | 0 (0/2) | 0 (0/2) | 0 (0/2) | 0 (0/2) | 50 (1/2) | 50 (1/2) | 50 (1/2) | 50 (1/2) | 0 (0/0) |  |  |
| Other | 60 (18/30) | 13 (4/30) | 6 (1/17) | 60 (3/5) | 13 (1/8) | 68 (21/31) | 70 (21/30) | 68 (21/31) | 55 (17/31) | 61 (11/18) | 77 (10/13) |  |  |
| *Acinetobacter spp* | |  |  |  |  |  |  |  |  |  |  |  |  |
| Overall | 56 (15/27) | 41 (11/27) | 55 (11/20) | 64 (7/11) | 50 (6/12) |  |  |  |  |  |  | 11 (3/27) |  |
| Blood | 67 (2/3) | 33 (1/3) | 50 (1/2) | 100 (1/1) | 50 (1/2) |  |  |  |  |  |  | 67 (2/3) |  |
| Other | 54 (13/24) | 42 (10/24) | 56 (10/18) | 60 (6/10) | 50 (5/10) |  |  |  |  |  |  | 4 (1/24) |  |
| *Enterobacter spp* | |  |  |  |  |  |  |  |  |  |  |  |  |
| Overall | 60 (15/25) | 44 (11/25) | 23 (3/13) | 60 (3/5) |  |  |  | 84 (21/25) | 42 (10/24) | 41 (7/17) | 13 (1/8) | 36 (8/22) |  |
| Blood | 44 (4/9) | 33 (3/9) | 0 (0/6) | 0 (0/1) |  |  |  | 89 (8/9) | 13 (1/8) | 25 (1/4) | 20 (1/5) | 0 (0/6) |  |
| Other | 69 (11/16) | 50 (8/16) | 43 (3/7) | 75 (3/4) |  |  |  | 81 (13/16) | 56 (9/16) | 46 (6/13) | 0 (0/3) | 50 (8/16) |  |

Data are expressed as % (n/N). Abbreviations: AMK = amikacin, CAZ = ceftazidime, CIP = ciprofloxacin, CRO = ceftriaxone, CTX = cefotaxime, DOR = doripenem, GEN = gentamicin, IPM = imipenem, LVX = levofloxacin, MEM = meropenem, SXT = trimethoprim-sulphamethoxazole, TGC = tigecycline, TZP = piperacillin/tazobactam.

**Table S2:** Proportion of resistance among gram-positive isolates overall.

|  | **Fluoro-quinolones** | **Sulfo-**  **namides** | **Penicillins** | | **Glyco-**  **peptides** | **Linco**  **samides** | **Macrolides** | **Oxazo-**  **lidinones** |
| --- | --- | --- | --- | --- | --- | --- | --- | --- |
|  | CIP | SXT | OXA | AMP | VAN | CLI | ERY | LZD |
| *S. aureus* |  |  |  |  |  |  |  |  |
| Overall | 22 (2/9) | 0 (0/5) | 36 (4/11) |  | 0 (0/10) | 40 (2/5) | 25 (2/8) | 0 (0/9) |
| Blood | 0 (0/1) | 0 (0/1) | 0 (0/1) |  | 0 (0/1) | 0 (0/1) | 0 (0/1) | 0 (0/1) |
| Other | 25 (2/8) | 0 (0/4) | 40 (4/10) |  | 0 (0/9) | 50 (2/4) | 29 (2/7) | 0 (0/8) |
| *E. faecalis* |  |  |  |  |  |  |  |  |
| Overall |  |  |  | 50 (6/12) | 13 (2/15) |  |  |  |
| Blood |  |  |  | 0 (0/1) | 0 (0/1) |  |  |  |
| Other |  |  |  | 54 (6/11) | 14 (2/14) |  |  |  |

Data are expressed as % (n/N). Abbreviations: AMP = ampicillin, CIP = ciprofloxacin, CLI = clindamycin, ERY = erythromycin, LZD = linezolid, OXA = oxacillin, SXT = trimethoprim-sulphamethoxazole, VAN = vancomycin.

**Table S3:** Proportion of resistance among gram-negative isolates in community-acquired infections

|  | **Aminoglycosides** | | **Carbapenems** | | | **3^rd^ generation cephalosporins** | | | **Fluoroquinolones** | | **Sulfo-**  **namides** | **Glycyl-**  **cyclins** | **Penicillins** |
| --- | --- | --- | --- | --- | --- | --- | --- | --- | --- | --- | --- | --- | --- |
|  | GEN | AMK | MEM | IPM | DOR | CRO | CTX | CAZ | CIP | LVX | SXT | TGC | TZP |
| *K. pneumoniae* | |  |  |  |  |  |  |  |  |  |  |  |  |
| Overall | 54 (7/13) | 15 (2/13) | 17 (1/6) | 25 (1/4) | 17 (1/6) | 62 (8/13) | 58 (7/12) | 54 (7/13) | 46 (6/13) | 30 (3/10) | 50 (2/4) |  |  |
| Blood | 33 (1/3) | 0 (0/3) | 0 (0/1) | - | 0 (0/1) | 33 (1/3) | 33 (1/3) | 33 (1/3) | 33 (1/3) | 0 (0/2) | 50 (1/2) |  |  |
| Other | 60 (6/10) | 20 (2/10) | 20 (1/5) | 25 (1/4) | 20 (1/5) | 70 (7/10) | 67 (6/9) | 60 (6/10) | 5 (5/10) | 38 (3/8) | 50 (1/2) |  |  |
| *P. aeruginosa* | |  |  |  |  |  |  |  |  |  |  |  |  |
| Overall | 45 (5/11) | 18 (2/11) | 33 (1/3) | 50 (1/2) | 29 (2/7) |  |  | 36 (4/11) | 36 (4/11) | 33 (3/9) |  |  | 44 (4/9) |
| Blood | 0 (0/2) | 0 (0/2) | 0 (0/2) | 0 (0/2) | 0 (0/2) |  |  | 0 (0/2) | 0 (0/2) | 0 (0/2) |  |  | 50 (1/2) |
| Other | 56 (5/9) | 22 (2/9) | 33 (1/3) | 50 (1/2) | 40 (2/5) |  |  | 44 (4/9) | 44 (4/9) | 43 (3/7) |  |  | 43 (3/7) |
| *E. coli* |  |  |  |  |  |  |  |  |  |  |  |  |  |
| Overall | 50 (5/10) | 10 (1/10) | 0 (0/3) | 0 (0/2) | 0 (0/3) | 45 (5/11) | 55 (6/11) | 45 (5/11) | 55 (6/11) | 56 (5/9) | 100 (2/2) |  |  |
| Blood | 0 (0/1) | 0 (0/1) | 0 (0/1) | 0 (0/1) | 0 (0/1) | 0 (0/1) | 100 (1/1) | 100 (1/1) | 0 (0/1) | 0 (0/1) | - |  |  |
| Other | 56 (5/9) | 11 (1/9) | 0 (0/2) | 0 (0/1) | 0 (0/2) | 50 (5/10) | 50 (5/10) | 40 (4/10) | 60 (6/10) | 63 (5/8) | 100 (2/2) |  |  |
| *Acinetobacter spp* | |  |  |  |  |  |  |  |  |  |  |  |  |
| Overall | 40 (2/5) | 0 (0/5) | 33 (1/3) | 0 (0/1) | 0 (0/2) |  |  |  |  |  |  | 0 (0/5) |  |
| Blood | - | - | - | - | - |  |  |  |  |  |  | - |  |
| Other | 40 (2/5) | 0 (0/5) | 33 (1/3) | 0 (0/1) | 0 (0/2) |  |  |  |  |  |  | 0 (0/5) |  |
| *Enterobacter spp* | |  |  |  |  |  |  |  |  |  |  |  |  |
| Overall | 50 (2/4) | 50 (2/4) | 0 (0/2) | 0 (0/1) |  |  |  | 75 (3/4) | 25 (1/4) | 33 (1/3) | 0 (0/1) | 25 (1/4) |  |
| Blood | 50 (1/2) | 50 (1/2) | 0 (0/2) | 0 (0/1) |  |  |  | 100 (2/2) | 0 (0/2) | 0 (0/1) | 0 (0/1) | 0 (0/2) |  |
| Other | 50 (1/2) | 50 (1/2) | - | - |  |  |  | 50 (1/2) | 50 (1/2) | 50 (1/2) | - | 50 (1/2) |  |

Data are expressed as % (n/N). Abbreviations: AMK = amikacin, CAZ = ceftazidime, CIP = ciprofloxacin, CRO = ceftriaxone, CTX = cefotaxime, DOR = doripenem, GEN = gentamicin, IPM = imipenem, LVX = levofloxacin, MEM = meropenem, SXT = trimethoprim-sulphamethoxazole, TGC = tigecycline, TZP = piperacillin/tazobactam.

**Table S4:** Proportion of resistance among gram-positive isolates in community-acquired infections

|  | **Fluoro-quinolones** | **Sulfo-**  **namides** | **Penicillins** | | **Glyco-**  **peptides** | **Linco**  **samides** | **Macrolides** | **Oxazo-**  **lidinones** |
| --- | --- | --- | --- | --- | --- | --- | --- | --- |
|  | CIP | SXT | OXA | AMP | VAN | CLI | ERY | LZD |
| *S. aureus* |  |  |  |  |  |  |  |  |
| Overall | 0 (0/4) | 0 (0/2) | 50 (1/2) |  | 0 (0/4) | 0 (0/2) | 0 (0/4) | 0 (0/4) |
| Blood | 0 (0/1) | 0 (0/1) | 0 (0/1) |  | 0 (0/1) | 0 (0/1) | 0 (0/1) | 0 (0/1) |
| Other | 0 (0/3) | 0 (0/1) | 100 (1/1) |  | 0 (0/3) | 0 (0/1) | 0 (0/3) | 0 (0/3) |
| *E. faecalis* |  |  |  |  |  |  |  |  |
| Overall |  |  |  | 33 (2/6) | 0 (0/7) |  |  |  |
| Blood |  |  |  | 0 (0/1) | 0 (0/1) |  |  |  |
| Other |  |  |  | 20 (2/5) | 0 (0/6) |  |  |  |

Data are expressed as % (n/N). Abbreviations: AMP = ampicillin, CIP = ciprofloxacin, CLI = clindamycin, ERY = erythromycin, LZD = linezolid, OXA = oxacillin, SXT = trimethoprim-sulphamethoxazole, VAN = vancomycin.

**Table S5:** Proportion of resistance among gram-negative isolates in hospital-acquired infections

|  | **Aminoglycosides** | | **Carbapenems** | | | **3^rd^ generation cephalosporins** | | | **Fluoroquinolones** | | **Sulfo-**  **namides** | **Glycyl-**  **cyclins** | **Penicillins** |
| --- | --- | --- | --- | --- | --- | --- | --- | --- | --- | --- | --- | --- | --- |
|  | GEN | AMK | MEM | IPM | DOR | CRO | CTX | CAZ | CIP | LVX | SXT | TGC | TZP |
| *K. pneumoniae* | |  |  |  |  |  |  |  |  |  |  |  |  |
| Overall | 59 (26/44) | 33 (15/45) | 32 (8/25) | 23 (3/13) | 24 (7/29) | 78 (39/50) | 78 (38/49) | 74 (37/50) | 66 (33/50) | 54 (20/37) | 57 (8/14) |  |  |
| Blood | 80 (4/5) | 20 (1/5) | 20 (1/5) | 25 (1/4) | 25 (1/4) | 100 (6/6) | 100 (6/6) | 100 (6/6) | 67 (4/6) | 40 (2/5) | 100 (1/1) |  |  |
| Other | 56 (22/39) | 35 (14/40) | 35 (7/20) | 22 (2/9) | 24 (6/25) | 75 (33/44) | 74 (32/43) | 70 (31/44) | 66 (29/44) | 56 (18/32) | 54 (7/13) |  |  |
| *P. aeruginosa* | |  |  |  |  |  |  |  |  |  |  |  |  |
| Overall | 39 (9/23) | 26 (6/23) | 11 (1/9) | 17 (1/6) | 31 (5/16) |  |  | 43 (10/23) | 26 (6/23) | 35 (7/20) |  |  | 48 (10/21) |
| Blood | - | - | - | - | - |  |  | - | - | - |  |  | - |
| Other | 39 (9/23) | 26 (6/23) | 11 (1/9) | 17 (1/6) | 31 (5/16) |  |  | 43 (10/23) | 26 (6/23) | 35 (7/20) |  |  | 48 (10/21) |
| *E. coli* |  |  |  |  |  |  |  |  |  |  |  |  |  |
| Overall | 59 (13/22) | 14 (3/22) | 6 (1/16) | 60 (3/5) | 14 (1/7) | 73 (16/22) | 76 (16/21) | 77 (17/22) | 55 (12/22) | 64 (7/11) | 73 (8/11) |  |  |
| Blood | 0 (0/1) | 0 (0/1) | 0 (0/1) | 0 (0/1) | 0 (0/1) | 0 (0/1) | 0 (0/1) | 0 (0/1) | 100 (1/1) | 100 (1/1) | - |  |  |
| Other | 62 (13/21) | 14 (3/21) | 7 (1/15) | 75 (3/4) | 17 (1/6) | 76 (16/21) | 80 (16/20) | 81 (17/21) | 52 (11/21) | 60 (6/10) | 73 (8/11) |  |  |
| *Acinetobacter spp* | |  |  |  |  |  |  |  |  |  |  |  |  |
| Overall | 59 (13/22) | 50 (11/22) | 59 (10/17) | 70 (7/10) | 60 (6/10) |  |  |  |  |  |  | 14 (3/22) |  |
| Blood | 67 (2/3) | 33 (1/3) | 50 (1/2) | 100 (1/1) | 50 (1/2) |  |  |  |  |  |  | 67 (2/3) |  |
| Other | 58 (11/19) | 53 (10/19) | 60 (9/15) | 67 (6/9) | 62 (5/8) |  |  |  |  |  |  | 5 (1/19) |  |
| *Enterobacter spp* | |  |  |  |  |  |  |  |  |  |  |  |  |
| Overall | 62 (13/21) | 43 (9/21) | 27 (3/11) | 75 (3/4) |  |  |  | 86 (18/21) | 45 (9/20) | 43 (6/14) | 14 (1/7) | 39 (7/18) |  |
| Blood | 43 (3/7) | 29 (2/7) | 0 (0/4) | - |  |  |  | 86 (6/7) | 17 (1/6) | 33 (1/3) | 25 (1/4) | 0 (0/4) |  |
| Other | 71 (10/14) | 50 (7/14) | 43 (3/7) | 75 (3/4) |  |  |  | 86 (12/14) | 57 (8/14) | 45 (5/11) | 0 (0/3) | 50 (7/14) |  |

Data are expressed as % (n/N). Abbreviations: AMK = amikacin, CAZ = ceftazidime, CIP = ciprofloxacin, CRO = ceftriaxone, CTX = cefotaxime, DOR = doripenem, GEN = gentamicin, IPM = imipenem, LVX = levofloxacin, MEM = meropenem, SXT = trimethoprim-sulphamethoxazole, TGC = tigecycline, TZP = piperacillin/tazobactam.

**Table S6:** Proportion of resistance among gram-positive isolates in hospital-acquired infections

|  | **Fluoro-quinolones** | **Sulfo-**  **namides** | **Penicillins** | | **Glyco-**  **peptides** | **Linco**  **samides** | **Macrolides** | **Oxazo-**  **lidinones** |
| --- | --- | --- | --- | --- | --- | --- | --- | --- |
|  | CIP | SXT | OXA | AMP | VAN | CLI | ERY | LZD |
| *S. aureus* |  |  |  |  |  |  |  |  |
| Overall | 40 (2/5) | 0 (0/3) | 33 (3/9) |  | 0 (0/6) | 66 (2/3) | 50 (2/4) | 0 (0/5) |
| Blood | - | - | - |  | - | - | - | - |
| Other | 40 (2/5) | 0 (0/3) | 33 (3/9) |  | 0 (0/6) | 66 (2/3) | 50 (2/4) | 0 (0/5) |
| *E. faecalis* |  |  |  |  |  |  |  |  |
| Overall |  |  |  | 66 (4/6) | 25 (2/8) |  |  |  |
| Blood |  |  |  | - | - |  |  |  |
| Other |  |  |  | 66 (4/6) | 25 (2/8) |  |  |  |

Data are expressed as % (n/N). Abbreviations: AMP = ampicillin, CIP = ciprofloxacin, CLI = clindamycin, ERY = erythromycin, LZD = linezolid, OXA = oxacillin, SXT = trimethoprim-sulphamethoxazole, VAN = vancomycin.

**Figure S1:** Study flowchart

**Figure S2:** Proportions of patients in whom one or more bacterial cultures were performed, by clinical syndrome.

Abbreviations: GI, gastrointestinal; IA, intra-abdominal; SST, skin and soft tissue infections; UTI, urinary tract infection.

**Figure S3:** Number of days from sample collection to reporting positive culture results, by specimen type.

**Figure S4:** Percentage of isolated bacteria, stratified by hospital ownership.

The numbers in bars indicate the number of isolates. The category ‘Other’ comprised *Serratia marcescens* (n=5), *Klebsiella oxytoca*, *Proteus mirabilis*, viridans group streptococci (each n=3), *Vibrio vulnificus*, *Moraxella* *catarrhalis*, *Micrococcus* *luteus* (each n=2), and *Stenotrophomonas* *maltophilia*, *Citrobacter* *freundii*, *Streptococcus* *mitis*, *Pseudomonas* *putida*, *Enterococcus* *avium*, *Morganella* *morganii* and *Proteus* *vulgaris* (each n=1).

Abbreviations: CoNS = coagulase-negative staphylococci.
